# Cumulative average triglyceride glucose-waist height index and incident cardiovascular disease in middle-aged and older adults: A nationwide cohort study from the china health and retirement longitudinal study

**DOI:** 10.1371/journal.pone.0333827

**Published:** 2026-02-26

**Authors:** Liang Zeng, Li Zhao, Jixiang Wan, Linji Li, Yiping Guo

**Affiliations:** 1 Department of Anesthesiology, Beijing Anzhen Nanchong Hospital of Capital Medical University & Nanchong Central Hospital, Nanchong, China; 2 Department of Anesthesiology, ZiYang Central Hospital, ZiYang, China; 3 Nanchong Center for Disease Control and Prevention, Nanchong, China; Xi’an Jiaotong University, CHINA

## Abstract

**Background:**

Insulin resistance (IR) is a significant risk factor for cardiovascular disease (CVD), yet practical biomarkers for long-term IR assessment are limited. The triglyceride glucose-waist-to-height ratio (TyG-WHtR) index, integrating lipid/glucose metabolism and central obesity, offers a novel composite marker. We investigated the association between cumulative average TyG-WHtR and incident CVD in middle-aged and older adults.

**Methods:**

This prospective cohort study utilized data from the China Health and Retirement Longitudinal Study (CHARLS). Participants aged ≥45 years without baseline CVD were included (n = 5,328). Cumulative average TyG-WHtR was calculated from Wave 1 (2011) and Wave 3 (2015) using the formula: [TyG × WHtR], where TyG = ln[(TG mg/dL × FBG mg/dL)/2] and WHtR = waist circumference (cm)/height (cm). Incident CVD was defined as new self-reported physician-diagnosed heart disease/stroke or active treatment during follow-up. Multivariable logistic regression and restricted cubic spline models assessed associations, adjusting for demographics, lifestyle, cardiometabolic risk factors, and comorbidities.

**Results:**

Over 4 years, 568 (10.7%) participants developed CVD. Higher cumulative average TyG-WHtR quartiles showed progressively increased CVD incidence (Q1: 7.4%, Q4: 13.3%; P-trend<0.001). After full adjustment, participants in Q2–Q4 had significantly higher CVD risk versus Q1 (Q2: OR=1.451, 95% CI: 1.095–1.928; Q3: OR=1.427, 1.066–1.917; Q4: OR=1.436, 1.035–2.000). Each 1-SD increase in TyG-WHtR was associated with a 18.3% higher CVD risk (OR=1.183, 95% CI: 1.052–1.332). A linear dose-response relationship was observed (P for overall = 0.018, P for nonlinear = 0.409), particularly for heart disease (P for overall = 0.010). Results remained consistent across subgroups (age, sex, smoking, comorbidities) and sensitivity analyses.

**Conclusions:**

Cumulative average TyG-WHtR independently predicts incident CVD in middle-aged and older Chinese adults. The cumulative average TyG-WHtR index may serve as a potential practical tool for early identification of individuals at elevated cardiovascular risk.

## Background

Cardiovascular disease (CVD) remains a primary global health threat among middle-aged and older populations, with persistently rising morbidity and mortality posing critical challenges for public health. According to the World Health Organization (WHO), CVD accounts for approximately 17.9 million annual deaths worldwide, representing 32% of all-cause mortality, with middle-aged and older adults constituting the most affected demographic [1,2]. In China, accelerated population aging has exacerbated the CVD burden, imposing substantial economic and psychosocial strains on families and society [3,4].

Chronically, insulin resistance (IR), a core pathophysiological mechanism underlying metabolic syndrome, is an established risk factor for CVD. IR-driven hyperinsulinemia, dysglycemia, dyslipidemia, and chronic inflammation collectively accelerate atherogenesis, thereby elevating cardiovascular risk [5–7]. While the hyperinsulinemic-euglycemic clamp remains the gold standard for IR assessment, its operational complexity and high cost limit large-scale clinical implementation [8]. Consequently, identifying simple yet reliable surrogate markers of IR is imperative for early CVD prevention and intervention.

The triglyceride-glucose index (TyG), calculated from fasting triglyceride and glucose levels, has emerged as a promising biomarker for IR evaluation and CVD risk stratification [9,10]. Simultaneously, the waist-to-height ratio (WHtR), a simplified measure of central obesity, demonstrates independent associations with CVD risk. Compared to conventional adiposity indices (e.g., waist circumference or body mass index), WHtR demonstrates superior discriminatory ability for CVD risk prediction [11,12]. Integrating TyG with WHtR to form the triglyceride glucose-waist-to-height ratio index (TyG-WHtR) may thus offer a novel composite biomarker for CVD prediction.

Nevertheless, evidence linking TyG-WHtR to CVD, particularly regarding long-term cumulative exposure in middle-aged and older populations, remains scarce. To address this gap, we leveraged data from a nationally representative cohort to investigate the association between cumulative average TyG-WHtR and incident CVD among middle-aged and older Chinese adults. This study aims to provide new scientific insights for early CVD risk assessment and preventive strategies.

## Methods

### Data source and study population

All data were derived from the China Health and Retirement Longitudinal Study (CHARLS), a nationally representative longitudinal cohort focusing on middle-aged and older adults (≥45 years). The baseline national survey (Wave 1) was conducted in 2011, encompassing 17,708 participants from 10,257 households across 150 counties/districts and 450 villages/residential communities. Follow-up surveys were conducted biennially (Wave 2: 2013; Wave 3: 2015; Wave 4: 2018). Venous blood samples were collected at Waves 1 and 3 (n = 11,847 and n = 13,420 participants, respectively). Detailed methodologies regarding sampling procedures, anthropometric measurements, and biomarker assessment have been previously published [13,14].

**Fig 1:**
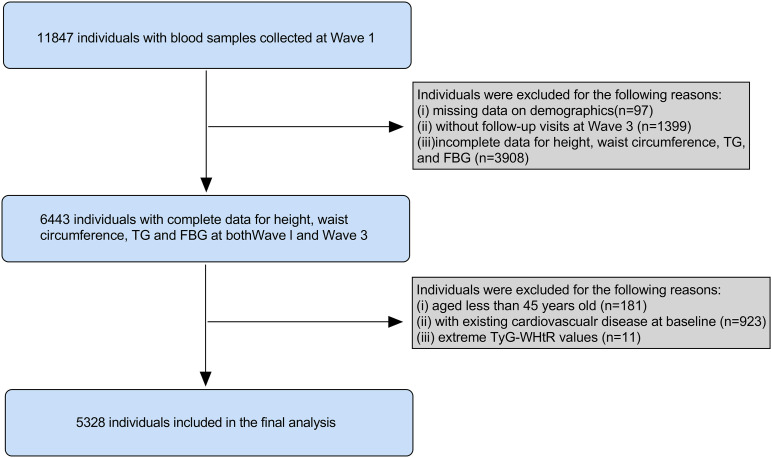
Flowchart of the study population. TG, triglyceride; FBG, fasting blood glucose; TyG-WHtR, triglyceride glucose-waist height ratio.

Given data availability, we utilized Wave 1 (2011) and Wave 3 (2015) datasets. From the initial 11,847 participants with baseline blood samples, we excluded those with: missing demographic data (n = 97), no Wave 3 follow-up (n = 1,399), incomplete height/waist circumference/triglyceride (TG)/fasting blood glucose (FBG) data at either wave (n = 3,908), age < 45 years (n = 181), extreme TyG-WHtR values (±3 SD from mean, n = 11), or pre-existing CVD at baseline (n = 923). The final analytical cohort comprised 5,328 participants (Fig 1).

The CHARLS study received ethical approval from the Biomedical Ethics Review Board of Peking University (IRB00001052–11015). All participants provided written informed consent. This study adheres to the STROBE (Strengthening the Reporting of Observational Studies in Epidemiology) guidelines [15].

### Data assessment and definitions

#### Exposure assessment.

The primary exposure was the cumulative average TyG-WHtR, calculated as:

TyG=ln[(Triglycerides (mg/dL)×Fasting glucose (mg/dL))/2].

WHtR = Waist circumference (cm)/Height (cm). TyG-WHtR = TyG × WHtR. Cumulative average TyG-WHtR was derived using the formula: (TyG-WHtR_2011_ + TyG-WHtR_2015_)/2

#### Outcome assessment.

Incident CVD was ascertained through standardized questionnaires at each survey wave. Participants were asked: “Have you been diagnosed with heart disease (myocardial infarction, coronary heart disease, angina, congestive heart failure) or stroke by a physician?” and “Are you currently receiving treatment for these conditions?” Consistent with established CHARLS methodology [16,17], CVD cases were defined by either: (1) Self-reported physician diagnosis, or (2) Active treatment for heart disease/stroke.

### Data collection

The following data were collected for this study: (i) demographics: gender, age, education level, place of residence (hukou), and marital status; (ii) physical measurements: systolic blood pressure (SBP), diastolic blood pressure (DBP), body mass index (BMI), waist circumference, and height; (iii) lifestyle information: smoking status and alcohol consumption; (iv) medical history: hypertension, diabetes mellitus, renal disorders, liver diseases, dyslipidemia, dyslipidemia medication history, hypertension medication history and diabetes medication history; (v) laboratory tests: glycosylated hemoglobin (HbA1c), FBG, TG, total cholesterol (TC), high-density lipoprotein cholesterol (HDL-c), low-density lipoprotein cholesterol (LDL-c).

### Handling of missing data

The distribution of missing data is shown in S1 Fig. To maximize statistical power while addressing missingness, we implemented multiple imputation using chained equations (MICE package in R) for variables with <10% missingness.

### Statistical analysis

Participants were categorized into quartiles (Q1-Q4) based on cumulative average TyG-WHtR values. Continuous variables are expressed as mean ± standard deviation (SD) with between-group comparisons assessed using one-way ANOVA, while categorical variables are presented as frequencies (percentages) analyzed by Pearson’s chi-square test. Logistic regression models were constructed to evaluate the association between cumulative average TyG-WHtR and incident CVD, with results reported as odds ratios (ORs) and 95% confidence intervals (CIs). Three sequential adjustment models were implemented: Model 1 adjusted for age and sex; Model 2 additionally incorporated smoking status, alcohol status, SBP, DBP, HbA1c, HDL-c, and LDL-c; Model 3 adjusted for all covariates. Multicollinearity was evaluated through variance inflation factors (all VIFs < 5, see S1 Table). Restricted cubic splines (RCS) were employed to examine potential nonlinear relationships. Subgroup analyses assessed effect modification across strata of age (<60 vs. ≥ 60 years), sex, smoking status, alcohol use, hypertension, dyslipidemia, and diabetes. In the sensitivity analysis, we tested the stability of the results by reanalyzing the data after removing all missing covariate data. All statistical analyses were performed using R software version 4.4.1, and a two-sided p-value < 0.05 was considered statistically significant.

## Results

### Baseline characteristics of participants

The flowchart illustrating the study population screening is presented in Fig 1. A total of 5,328 participants were enrolled in our study, comprising 2,847 (53.4%) males and 2,481 (46.6%) females. The mean age of the participants was 58.5 years. The baseline characteristics of enrolled participants, categorized by quartiles of the cumulative average TyG-WHtR, are outlined in Table 1. Among all included participants, the mean (SD) cumulative average TyG-WHtR was 3.5 (0.6).

**Table 1:** Baseline characteristics of individuals classified by quartiles of the cumulative average TyG-WHtR.

		Quartiles of the cumulative average TyG-WHtR				
Characteristic	OverallN = 5328	Q1N = 1332	Q2N = 1332	Q3N = 1332	Q4N = 1332	P Value
Gender						<0.001
male	2,481 (47%)	881 (66%)	676 (51%)	547 (41%)	377 (28%)	
Female	2,847 (53%)	451 (34%)	656 (49%)	785 (59%)	955 (72%)	
Age, years	58.5 ± (8.7)	59.1 ± (8.9)	58.0 ± (8.7)	58.3 ± (8.6)	58.5 ± (8.5)	0.003
Education level						0.14
Elementary school or below	3,783 (71%)	941 (71%)	950 (71%)	918 (69%)	974 (73%)	
Middle school	1,414 (27%)	361 (27%)	348 (26%)	371 (28%)	334 (25%)	
College or above	131 (2.5%)	30 (2.3%)	34 (2.6%)	43 (3.2%)	24 (1.8%)	
Residence						<0.001
Urban	753 (14%)	125 (9.4%)	177 (13%)	234 (18%)	217 (16%)	
Rural	4,575 (86%)	1,207 (91%)	1,155 (87%)	1,098 (82%)	1,115 (84%)	
Marital status						>0.9
married	4,555 (85%)	1,138 (85%)	1,134 (85%)	1,146 (86%)	1,137 (85%)	
Others	773 (15%)	194 (15%)	198 (15%)	186 (14%)	195 (15%)	
SBP, mmHg	128.5 ± (20.8)	123.1 ± (19.6)	126.1 ± (20.2)	129.5 ± (20.1)	135.3 ± (21.1)	<0.001
DBP, mmHg	75.0 ± (12.0)	71.6 ± (11.7)	73.8 ± (11.8)	75.7 ± (11.5)	78.9 ± (12.0)	<0.001
BMI, kg/m2	23.5 ± (3.8)	20.6 ± (3.0)	22.2 ± (2.4)	24.1 ± (3.0)	26.9 ± (3.5)	<0.001
Smoking status						<0.001
Never	3,282 (62%)	600 (45%)	788 (59%)	895 (67%)	999 (75%)	
Former	409 (7.7%)	114 (8.6%)	94 (7.1%)	114 (8.6%)	87 (6.5%)	
Current	1,637 (31%)	618 (46%)	450 (34%)	323 (24%)	246 (18%)	
Drinking status						<0.001
Never	3,224 (61%)	665 (50%)	793 (60%)	834 (63%)	932 (70%)	
Former	416 (7.8%)	119 (8.9%)	88 (6.6%)	116 (8.7%)	93 (7.0%)	
Current	1,688 (32%)	548 (41%)	451 (34%)	382 (29%)	307 (23%)	
Dyslipidemia	441 (8.3%)	50 (3.8%)	70 (5.3%)	112 (8.4%)	209 (16%)	<0.001
Hypertension	1,219 (23%)	168 (13%)	216 (16%)	340 (26%)	495 (37%)	<0.001
Diabetes	285 (5.3%)	29 (2.2%)	41 (3.1%)	67 (5.0%)	148 (11%)	<0.001
kidney	264 (5.0%)	70 (5.3%)	75 (5.6%)	50 (3.8%)	69 (5.2%)	0.12
liver	149 (2.8%)	48 (3.6%)	29 (2.2%)	37 (2.8%)	35 (2.6%)	0.2
Dyslipidemia med history	212 (4.0%)	22 (1.7%)	27 (2.0%)	52 (3.9%)	111 (8.3%)	<0.001
Hypertension med history	864 (16%)	103 (7.7%)	142 (11%)	235 (18%)	384 (29%)	<0.001
Diabetes med history	164 (3.1%)	20 (1.5%)	25 (1.9%)	30 (2.3%)	89 (6.7%)	<0.001
TC, mg/dL	132.6 ± (110.6)	81.4 ± (43.9)	104.0 ± (68.6)	132.0 ± (75.6)	213.1 ± (163.3)	<0.001
HDL-c, mg/dL	51.4 ± (15.4)	59.3 ± (16.3)	54.0 ± (14.0)	49.8 ± (13.1)	42.5 ± (12.6)	<0.001
LDL-c, mg/dL	116.1 ± (34.7)	108.9 ± (30.7)	116.1 ± (31.1)	121.8 ± (33.9)	117.5 ± (40.9)	<0.001
TyG	8.7 ± (0.7)	8.2 ± (0.5)	8.5 ± (0.5)	8.7 ± (0.5)	9.3 ± (0.7)	<0.001
TyG-WHtR	3.5 ± (0.6)	2.80 ± (0.3)	3.24 ± (0.1)	3.62 ± (0.1)	4.21± (0.3)	<0.001

SBP, systolic blood pressure; DBP, diastolic blood pressure; BMI, body mass index; FBG, fasting blood glucose; HbA1c, glycosylated hemoglobin A1c; TG, triglyceride; TC, total cholesterol; HDL‐c, high‐density lipoprotein cholesterol; LDL-c, low-density lipoprotein cholesterol; TyG, triglyceride glucose index; TyG-WHtR, triglyceride glucose-waist height ratio.

Compared to the lowest quartile group (Q1), participants in groups with higher levels of cumulative average TyG-WHtR (Q2-Q4) were slightly younger, exhibited a greater proportion of females and urban residents, and had higher values of SBP, DBP, TC, LDL-c (Q2-Q3), and BMI, but lower HDL-c values (all P-values < 0.05). Furthermore, they showed a higher prevalence of hypertension, dyslipidemia, and diabetes (all P-values < 0.05). Notably, the prevalence of current smoking and alcohol consumption decreased progressively across quartiles (Q1 to Q4), while the proportion of never-smokers and never-drinkers increased significantly (Table 1). However, no statistically significant differences in the prevalence of liver or kidney diseases, marital status, or education level were observed across all groups (all P-values > 0.05).

### Association between the cumulative average TyG-WHtR and cardiovascular disease incidence

**Fig 2:**
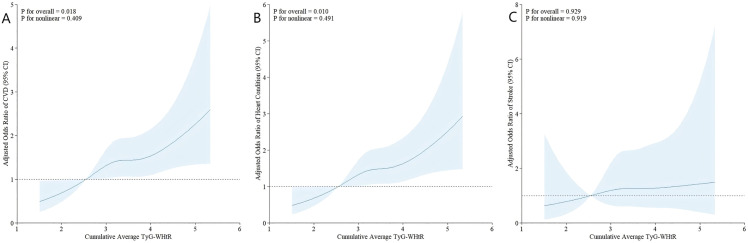
Association between cumulative average TyG-WHtR and CVD (A), incident heart disease (B), and incident stroke (C). The model was adjusted for all covariates. TyG-WHtR, triglyceride glucose-waist height ratio; CVD, Cardiovascular disease..

In total, 568 (10.7%) participants developed CVD during a 4-year follow-up (Wave 1 in 2011 to Wave 3 in 2015). As depicted in Fig 2, the incidence rates of CVD increased progressively across quartiles of the cumulative average TyG-WHtR (from Q1 to Q4), with 98 (7.4%), 138 (10.4%), 155 (11.6%), and 177 (13.3%) cases observed in four groups of participants, respectively (Table 2).

**Table 2:** Association between the cumulative average TyG-WHtR and CVD incidence.

Cumulative Average TyG-WHtR	Quartiles					Continuous
	Quartile 1	Quartile 2	Quartile 3	Quartile 4	P for trend	Per 1 SD increase
Median	2.80	3.24	3.62	4.21	–	–
Cases, n (%)	98 (7.4%)	138 (10.4%)	155 (11.6%)	177 (13.3%)	–	–
Crude, OR (95% CI)	Reference	1.455 (1.112–1.912)	1.658 (1.274–2.167)	1.930 (1.492–2.509)	< 0.001	1.285 (1.178–1.400)
Model 1, OR (95% CI)	Reference	1.471 (1.120–1.937)	1.624 (1.241–2.134)	1.829 (1.398–2.404)	< 0.001	1.256 (1.148–1.374)
Model 2, OR (95% CI)	Reference	1.458 (1.103–1.932)	1.531 (1.151–2.045)	1.677 (1.231–2.294)	0.002	1.224 (1.100–1.363)
Model 3, OR (95% CI)	Reference	1.450 (1.094–1.927)	1.412 (1.054–1.898)	1.436 (1.035–2.000)	0.047	1.183 (1.052–1.332)

Crude: No covariates were adjusted. Model 1, adjusted for age and gender; Model 2, adjusted for age, gender, smoking status, drinking status, SBP, DBP, HbA1c, HDL-c, LDL-c; Model 3, adjusted for all covariates. TyG-WHtR, triglyceride glucose-waist height ratio; CVD, Cardiovascular disease; OR, odds ratio; CI, confidence interval; SD, standard deviation.

After adjusting for multiple covariates, the fully adjusted logistic regression model indicated that higher levels of cumulative average TyG-WHtR (Q2-Q4) significantly increased the odds ratios for incident CVD compared to Q1 (OR 1.451, 95% CI 1.095–1.928 for Q2; OR 1.427, 95% CI 1.066–1.917 for Q3; OR 1.436, 95% CI 1.035–2.000 for Q4) (Table 2). Consistently, when considered as a continuous variable, each 1-SD increase in cumulative average TyG-WHtR was significantly associated with incident CVD (OR 1.183, 95% CI 1.052–1.332).

Furthermore, logistic regression analyses examining the correlation between cumulative average TyG-WHtR and the components of CVD (heart disease or stroke) revealed that cumulative average TyG-WHtR was significantly associated with incident heart disease (OR 1.456, 95% CI 1.081–1.967 for Q2; OR 1.432, 95% CI 1.052–1.958 for Q3; OR 1.550, 95% CI 1.098–2.197 for Q4; OR 1.229, 95% CI 1.086–1.393 per 1-SD increase), but not associated with incident stroke, as shown in Table 3.

**Table 3:** Association between the cumulative average TyG-WHtR and the components of CVD.

Cumulative Average TyG-WHtR	Quartiles					Continuous
	Quartile 1	Quartile 2	Quartile 3	Quartile 4	P for trend	Per 1 SD increase
Heart Condition						
Cases, n (%)	86 (6.5%)	122 (9.2%)	136 (10.2%)	158 (11.9%)	–	–
Crude, OR (95%CI)	Reference	1.461(1.098–1.951)	1.648 (1.246–2.189)	1.950 (1.486–2.575)	<0.001	1.289 (1.177–1.412)
Model 1, OR (95%CI)	Reference	1.446 (1.084–1.937)	1.559 (1.173–2.084)	1.757 (1.323–2.346)	<0.001	1.239 (1.126–1.362)
Model 2, OR (95%CI)	Reference	1.461 (1.089–1.969)	1.540 (1.139–2.092)	1.751 (1.264–2.437	<0.001	1.250 (1.117–1.400)
Model 3, OR (95%CI)	Reference	1.456 (1.081–1.967)	1.432 (1.052–1.958)	1.550 (1.098–2.197)	0.026	1.229 (1.086–1.393)
Stroke						
Cases, n (%)	15(1.1%)	19 (1.4%)	22 (1.7%)	26 (2%)	–	–
Crude, OR (95%CI)	Reference	1.271 (0.645–2.550)	1.475 (0.767–2.911)	1.748 (0.933–3.394)	0.074	1.242 (1.001–1.537)
Model 1, OR (95%CI)	Reference	1.445 (0.731–2.909)	1.800 (0.928–3.587	2.358 (1.224–4.695)	0.009	1.383 (1.103–1.728)
Model 2, OR (95%CI)	Reference	1.351 (0.672–2.765)	1.389 (0.685–2.881	1.504 (0.701–3.309)	0.331	1.130 (0.868–1.474)
Model 3, OR (95%CI)	Reference	1.363 (0.677–2.793)	1.366 (0.667–2.861)	1.373 (0.612–3.139)	0.477	1.086 (0.815–1.453)

Crude: No covariates were adjusted. Model 1, adjusted for age and gender; Model 2, adjusted for age, gender, smoking status, drinking status, SBP, DBP, HbA1c, HDL-c, LDL-c; Model 3, Corrected for all covariates. TyG-WHtR, triglyceride glucose-waist height ratio; OR, odds ratio; CI, confidence interval; SD, standard deviation.

In comparative analyses, neither quartiles of TyG nor per 1-SD increase in TyG predicted CVD risk after full adjustment (Q4 OR: 1.011, 95% CI: 0.748–1.366; per SD OR: 1.059, 95% CI: 0.931–1.204). WHtR alone showed significant associations (Q4 OR: 1.429, 95% CI: 1.093–1.874; per SD OR: 1.140, 95% CI: 1.057–1.237), but effect sizes were consistently attenuated versus the combined TyG-WHtR index (S2 Table and S3 Table).

Additionally, Cox proportional hazards analysis investigating the association between TyG-WHtR (assessed exclusively at wave 1) and CVD incidence during waves 2–4 revealed consistent results: for the fourth quartile (Q4), the hazard ratio was 1.380 (95% CI: 1.147–1.661); with each 1-SD increase in TyG-WHtR, the hazard ratio was 1.154 (S4 Table).

The fully adjusted restricted cubic spline (RCS) regression model indicated a positive linear correlation between cumulative average TyG-WHtR and incident CVD (P for overall = 0.018, P for nonlinear = 0.409) (Fig 2A). Additionally, the RCS model demonstrated a linear relationship between cumulative average TyG-WHtR and incident heart disease (P for overall = 0.010, P for nonlinear = 0.491) (Fig 2B). However, cumulative average TyG-WHtR did not show a significant association with incident stroke in our analysis (P for overall = 0.929) (Fig 2C).

### Subgroup analyses and sensitivity analyses

**Fig 3:**
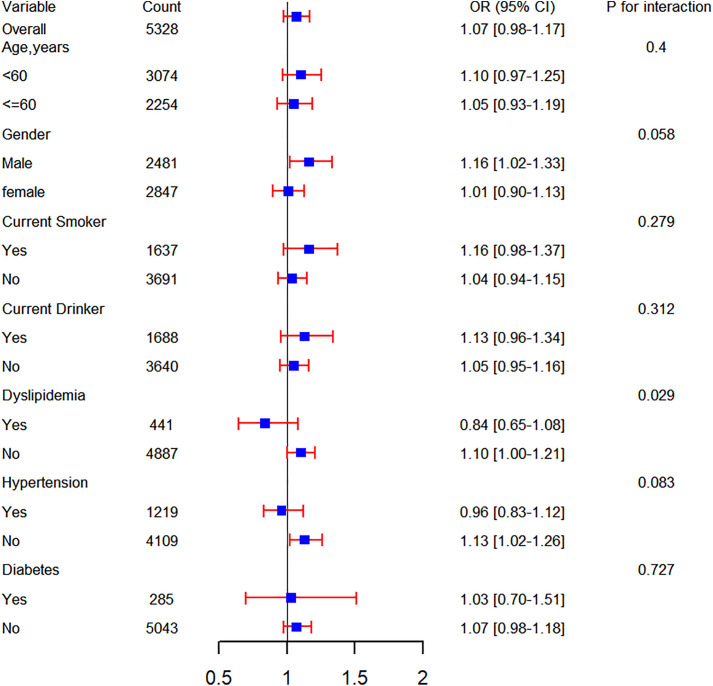
Subgroup analyses of the association between the cumulative average TyG-WHtR and CVD incidence. TyG-WHtR, triglyceride glucose-waist height ratio; CVD, Cardiovascular disease; OR, odds ratio; CI, confidence interval.

To further explore the relationship between cumulative average TyG-WHtR and the incidence of cardiovascular disease (CVD), a series of subgroup analyses were conducted. As shown in Fig 3, no significant effect modification was observed in any subgroup, including age, gender, current smokers, current drinkers, and the prevalence of dyslipidemia, hypertension, or diabetes (all interaction P values > 0.05). In addition, cumulative average TyG-WHtR showed no significant association with increased cardiovascular disease risk in these subgroups: adults ≥60 years, females, non-smokers, alcohol drinkers, hypertensive patients, dyslipidemic individuals, or diabetic patients.

Furthermore, the positive correlation between cumulative average TyG-WHtR and CVD incidence remained consistent in the fully adjusted model after excluding participants with any missing values of covariates (OR 1.475, 95% CI 1.099–1.987 for Q2; OR 1.541, 95% CI 1.139–2.094 for Q3; OR 1.579, 95% CI 1.122–2.231 for Q4; OR 1.228, 95% CI 1.087–1.388 per 1-SD increase) (S5 Table).

## Discussion

This prospective nationwide cohort study, based on 4-year follow-up data from a representative sample, is the first to systematically evaluate the association between the cumulative average triglyceride glucose-waist height ratio index (TyG-WHtR) and the risk of cardiovascular disease (CVD) incidence among middle-aged and older Chinese adults, encompassing 5,328 participants. The results revealed that each 1-SD increase in the cumulative average TyG-WHtR index was associated with a 18.3% increase in CVD risk (OR = 1.183, 95% CI: 1.052–1.332), and showed a linear dose-response relationship with incident heart disease.

CVD remains one of the leading global health burdens, imposing significant economic and resource pressures on healthcare systems worldwide. Notably, the burden of CVD is substantially higher in low- and middle-income countries compared to high-income countries, primarily attributed to factors such as accelerated population aging, the epidemic of metabolic diseases, and uneven distribution of medical resources [18]. Insulin resistance (IR), a core pathophysiological state characterized by reduced sensitivity of peripheral tissues (such as skeletal muscle, adipose tissue, and liver) to insulin-mediated glucose metabolism regulation [19], has been extensively demonstrated as a key driver of atherosclerosis and independently increases CVD risk beyond traditional risk factors like hypertension and dyslipidemia [20–22]. Although the hyperinsulinemic-euglycemic clamp test is the gold standard for detecting IR, its technical complexity and high cost limit its widespread application in clinical practice. Therefore, there is an urgent need to develop simple and reliable surrogate markers for IR to facilitate early prevention and control of CVD.

Studies by Liu et al. [23] and Zhang et al. [24] on young diabetic patients in the United States found a significant association between the TyG index and all-cause mortality as well as CVD. Furthermore, a meta-analysis of 31 studies by Xue et al. found that abdominal obesity was associated with an increased risk of CVD, and WHtR was a good predictor of CVD [25]. A cohort study by Zhang et al. found a positive correlation between WHtR and CVD incidence, indicating that WHtR can be used to predict CVD incidence in adults with hypertension [26]. The findings of this study revealed a significant increase in CVD incidence risk associated with rising levels of the cumulative average TyG-WHtR index. This finding aligns with previous research on the associations of the TyG index or WHtR individually with CVD risk but further emphasizes the combined cumulative effect when integrating both indicators [27]. The TyG index, as a surrogate marker for insulin resistance, reflects impaired insulin function in regulating glucose and lipid metabolism. Conversely, WHtR, a simple measure of abdominal obesity, is directly linked to central adiposity, a major risk factor for cardiovascular disease. Therefore, the cumulative average TyG-WHtR index may offer a more comprehensive reflection of the combined impact of insulin resistance and abdominal obesity on cardiovascular health. This is similar to Ren et al.‘s study, which showed that cumulative TyG-WHtR predicts CVD incidence [28]. in addition, TyG-WHtR outperformed its individual components: TyG alone did not independently predict CVD in our cohort after multivariable adjustment, while WHtR exhibited weaker effect sizes. This underscores that integrating lipid-glucose dysregulation (TyG) and abdominal obesity (WHtR) provides synergistic value for CVD risk stratification, capturing multidimensional metabolic dysfunction.

Consistent associations were observed across all subgroups stratified by age, gender, smoking, alcohol consumption, and comorbidities (e.g., hypertension, diabetes), indicating the predictive value of TyG-WHtR for the majority of individuals. This is consistent with findings from another study showing stable associations between the TyG index and CVD across different populations [29]. Our results significantly contribute to elucidating the relationship between the cumulative average TyG-WHtR and incident CVD, highlighting its value as an economical and valuable early indicator for identifying individuals predisposed to developing CVD.

Despite the strengths of this study, including its nationally representative sample and adjustment for multiple confounders, several limitations should be acknowledged. First, the diagnosis of CVD relied on self-reporting by participants, which may involve under-reporting or misreporting. However, this study excluded participants with pre-existing CVD at baseline and used standardized questionnaires for data collection, which may have reduced information bias to some extent. Second, although the cohort study design suggests temporal sequence, residual confounding cannot be entirely ruled out in observational studies. While we adjusted for antihypertensive, lipid-lowering, and antidiabetic medications, detailed information on other medications (e.g., diuretics) was not systematically collected in CHARLS. This represents a potential unmeasured confounder that should be addressed in future studies with more comprehensive medication data. Finally, our participants were exclusively from the middle-aged and older Chinese population; therefore, these findings may not be generalizable to other countries or younger age groups.

## Conclusion

In summary, this study demonstrates a significant positive association between the cumulative average TyG-WHtR index and the risk of incident cardiovascular disease among middle-aged and older Chinese adults. Each standard deviation increase in the cumulative average TyG-WHtR index was associated with a 18.3% increased risk of CVD (OR = 1.183, 95% CI: 1.052–1.332). These findings suggest the cumulative average TyG-WHtR index may serve as a potential practical tool for early identification of individuals at elevated cardiovascular risk.

## Supporting information

S1 Fig The distribution of missing data. (TIF)

S1 Table Collinearity Statistics. (DOCX)

S2 Table Association between the cumulative average TyG and CVD incidence. (DOCX)

S3 Table Association between the cumulative average WHtR and CVD incidence. (DOCX)

S4 Table Association Between TyG-WHtR at Wave 1 and Incident CVD Incidence from Wave 2 to Wave 4: Cox Regression Results. (DOCX)

S5 Table Association between the cumulative average TyG-WHtR and CVD incidence after excluding individuals with any missing value. (DOCX)

## Abbreviations

IR: Insulin resistance
CVD: Cardiovascular disease
TyG-WHtR: triglyceride glucose-waist height ratio
CHARLS: China health and retirement longitudinal study
TG: Triglyceride
FBG: Fasting blood glucose
BMI: Body mass index
RCS: Restricted cubic spline
SD: Standard deviation
OR: Odds ratio
CI: Confidence interval
STROBE: Strengthening the reporting of observational studies in epidemiology
SBP: Systolic blood pressure
DBP: Diastolic blood pressure
HbA1c: Glycosylated hemoglobin
TC: Total cholesterol
HDL-c: High-density lipoprotein cholesterol
LDL-c: Low-density lipoprotein cholesterol
Q1: Quartile 1
Q2: Quartile 2
Q3: Quartile 3
Q4: Quartile 4
VIF: Variance inflation factor

## References

[1] Vaduganathan M, Mensah GA, Turco JV, Fuster V, Roth GA. The Global Burden of Cardiovascular Diseases and Risk: A Compass for Future Health. Journal of the American College of Cardiology, 2022;80(25):2361–71. 36368511 10.1016/j.jacc.2022.11.005

[2] Roth GA, Mensah GA, Johnson CO, Addolorato G, Ammirati E, Baddour LM, et al. Global burden of cardiovascular diseases and risk factors, 1990-2019: update from the GBD 2019 study. Journal of the American College of Cardiology. 2020;76(25):2982–3021. 33309175 10.1016/j.jacc.2020.11.010 PMC7755038

[3] Liu M, Wang W, Zhou M. Trend analysis on the mortality of cardiovascular diseases from 2004 to 2010 in China. Zhonghua Liu Xing Bing Xue Za Zhi. 2013;34(10):985–8. 24377992

[4] Ma L, Chen W-W, Gao R-L, Liu L-S, Zhu M-L, Wang Y-J, et al. China cardiovascular diseases report 2018: an updated summary. Journal of Geriatric Cardiology. 2020;17(1):1–8. 32133031 10.11909/j.issn.1671-5411.2020.01.001 PMC7008101

[5] Mahajan R. Insulin Resistance: Quest for Surrogate Markers. Int J Appl Basic Med Res. 2017;7(3):149. doi: 10.4103/ijabmr.IJABMR_198_17 28904911 PMC5590374

[6] Duan M, Zhao X, Li S, Miao G, Bai L, Zhang Q, et al. Metabolic score for insulin resistance (METS-IR) predicts all-cause and cardiovascular mortality in the general population: evidence from NHANES 2001-2018. Cardiovasc Diabetol. 2024;23(1):243. doi: 10.1186/s12933-024-02334-8 38987779 PMC11238348

[7] Yin B, Wu Z, Xia Y, Xiao S, Chen L, Li Y. Non-linear association of atherogenic index of plasma with insulin resistance and type 2 diabetes: a cross-sectional study. Cardiovasc Diabetol. 2023;22(1):157. doi: 10.1186/s12933-023-01886-5 37386500 PMC10311747

[8] Ye J, Ye X, Jiang W, Lu C, Geng X, Zhao C, et al. Targeted lipidomics reveals associations between serum sphingolipids and insulin sensitivity measured by the hyperinsulinemic-euglycemic clamp. Diabetes Res Clin Pract. 2021;173:108699. doi: 10.1016/j.diabres.2021.108699 33592213

[9] Cui C, Qi Y, Song J, Shang X, Han T, Han N, et al. Comparison of triglyceride glucose index and modified triglyceride glucose indices in prediction of cardiovascular diseases in middle aged and older Chinese adults. Cardiovasc Diabetol. 2024;23(1):185. doi: 10.1186/s12933-024-02278-z 38812015 PMC11138075

[10] Cui C, Liu L, Zhang T, Fang L, Mo Z, Qi Y, et al. Triglyceride-glucose index, renal function and cardiovascular disease: a national cohort study. Cardiovasc Diabetol. 2023;22(1):325. doi: 10.1186/s12933-023-02055-4 38017519 PMC10685637

[11] Lo K, Huang Y-Q, Shen G, Huang J-Y, Liu L, Yu Y-L, et al. Effects of waist to height ratio, waist circumference, body mass index on the risk of chronic diseases, all-cause, cardiovascular and cancer mortality. Postgrad Med J. 2021;97(1147):306–11. doi: 10.1136/postgradmedj-2020-137542 32371408

[12] Ashwell M, Gunn P, Gibson S. Waist-to-height ratio is a better screening tool than waist circumference and BMI for adult cardiometabolic risk factors: systematic review and meta-analysis. Obes Rev. 2012;13(3):275–86. doi: 10.1111/j.1467-789X.2011.00952.x 22106927

[13] Chen X, Crimmins E, Hu PP, Kim JK, Meng Q, Strauss J, et al. Venous Blood-Based Biomarkers in the China Health and Retirement Longitudinal Study: Rationale, Design, and Results From the 2015 Wave. Am J Epidemiol. 2019;188(11):1871–7. doi: 10.1093/aje/kwz170 31364691 PMC6825825

[14] Zhao Y, Hu Y, Smith JP, Strauss J, Yang G. Cohort profile: the China Health and Retirement Longitudinal Study (CHARLS). Int J Epidemiol. 2014;43(1):61–8. doi: 10.1093/ije/dys203 23243115 PMC3937970

[15] von Elm E, Altman DG, Egger M, Pocock SJ, Gøtzsche PC, Vandenbroucke JP, et al. The Strengthening the Reporting of Observational Studies in Epidemiology (STROBE) statement: guidelines for reporting observational studies. Epidemiology. 2007;18(6):800–4. doi: 10.1097/EDE.0b013e3181577654 18049194

[16] Wu Y, Yang Y, Zhang J, Liu S, Zhuang W. The change of triglyceride-glucose index may predict incidence of stroke in the general population over 45 years old. Cardiovasc Diabetol. 2023;22(1):132. doi: 10.1186/s12933-023-01870-z 37296457 PMC10257314

[17] Gao K, Cao L-F, Ma W-Z, Gao Y-J, Luo M-S, Zhu J, et al. Association between sarcopenia and cardiovascular disease among middle-aged and older adults: Findings from the China health and retirement longitudinal study. EClinicalMedicine. 2022;44:101264. doi: 10.1016/j.eclinm.2021.101264 35059617 PMC8760427

[18] Crea F. The burden of cardiovascular risk factors: a global perspective. Eur Heart J. 2022;43(30):2817–20. doi: 10.1093/eurheartj/ehac430 35933110

[19] James DE, Stöckli J, Birnbaum MJ. The aetiology and molecular landscape of insulin resistance. Nat Rev Mol Cell Biol. 2021;22(11):751–71. doi: 10.1038/s41580-021-00390-6 34285405

[20] Hill MA, Yang Y, Zhang L, Sun Z, Jia G, Parrish AR, et al. Insulin resistance, cardiovascular stiffening and cardiovascular disease. Metabolism. 2021;119:154766. doi: 10.1016/j.metabol.2021.154766 33766485

[21] Horton WB, Love KM, Gregory JM, Liu Z, Barrett EJ. Metabolic and vascular insulin resistance: partners in the pathogenesis of cardiovascular disease in diabetes. Am J Physiol Heart Circ Physiol. 2025;328(6):H1218–36. doi: 10.1152/ajpheart.00826.2024 40257392 PMC12172477

[22] Bender SB, McGraw AP, Jaffe IZ, Sowers JR. Mineralocorticoid receptor-mediated vascular insulin resistance: an early contributor to diabetes-related vascular disease? Diabetes. 2013;62(2):313–9. doi: 10.2337/db12-0905 23349535 PMC3554383

[23] Liu C, Liang D, Xiao K, Xie L. Association between the triglyceride-glucose index and all-cause and CVD mortality in the young population with diabetes. Cardiovasc Diabetol. 2024;23(1):171. doi: 10.1186/s12933-024-02269-0 38755682 PMC11097545

[24] Zhang Q, Xiao S, Jiao X, Shen Y. The triglyceride-glucose index is a predictor for cardiovascular and all-cause mortality in CVD patients with diabetes or pre-diabetes: evidence from NHANES 2001-2018. Cardiovasc Diabetol. 2023;22(1):279. doi: 10.1186/s12933-023-02030-z 37848879 PMC10583314

[25] Xue R, Li Q, Geng Y, Wang H, Wang F, Zhang S. Abdominal obesity and risk of CVD: a dose-response meta-analysis of thirty-one prospective studies. Br J Nutr. 2021;126(9):1420–30. doi: 10.1017/S0007114521000064 33431092

[26] Zhang S, Fu X, Du Z, Guo X, Li Z, Sun G, et al. Is waist-to-height ratio the best predictive indicator of cardiovascular disease incidence in hypertensive adults? A cohort study. BMC Cardiovasc Disord. 2022;22(1):214. doi: 10.1186/s12872-022-02646-1 35545759 PMC9092683

[27] Xu J, Cai D, Jiao Y, Liao Y, Shen Y, Shen Y, et al. Insights into the complex relationship between triglyceride glucose-waist height ratio index, mean arterial pressure, and cardiovascular disease: a nationwide prospective cohort study. Cardiovasc Diabetol. 2025;24(1):93. doi: 10.1186/s12933-025-02657-0 40022080 PMC11871683

[28] Ren Q, Huang Y, Liu Q, Chu T, Li G, Wu Z. Association between triglyceride glucose-waist height ratio index and cardiovascular disease in middle-aged and older Chinese individuals: a nationwide cohort study. Cardiovasc Diabetol. 2024;23(1):247. doi: 10.1186/s12933-024-02336-6 38992634 PMC11241990

[29] Li F, Wang Y, Shi B, Sun S, Wang S, Pang S, et al. Association between the cumulative average triglyceride glucose-body mass index and cardiovascular disease incidence among the middle-aged and older population: a prospective nationwide cohort study in China. Cardiovasc Diabetol. 2024;23(1):16. doi: 10.1186/s12933-023-02114-w 38184577 PMC10771655

