# Supplementary figures and images for: Cumulative average triglyceride glucose-waist height index and incident cardiovascular disease in middle-aged and older adults: A nationwide cohort study from the china health and retirement longitudinal study
Source: PLoS One. 2026 Feb 26;21(2):e0333827. doi: 10.1371/journal.pone.0333827 (PMC12944753; doi:10.1371/journal.pone.0333827)

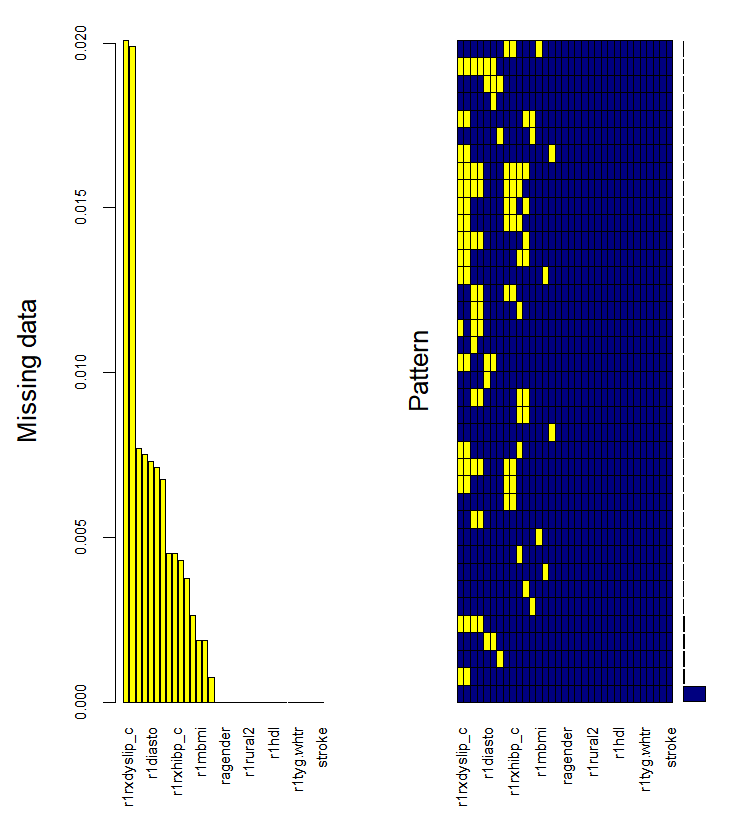

Supplement: S1 Fig — (TIF) [file pone.0333827.s001.tif]
