# Supplementary material for: Cumulative average triglyceride glucose-waist height index and incident cardiovascular disease in middle-aged and older adults: A nationwide cohort study from the china health and retirement longitudinal study
Source: PLoS One. 2026 Feb 26;21(2):e0333827. doi: 10.1371/journal.pone.0333827 (PMC12944753; doi:10.1371/journal.pone.0333827)
Supplement: S1 Table — (DOCX) [file pone.0333827.s002.docx]

S1 Table. Collinearity Statistics

| Variables | GVIF | Adj_VIF |
| --- | --- | --- |
| Age | 1.46648952112725 | 1.21098700287297 |
| Gender | 2.55201868943036 | 1.59750389340069 |
| Education Level | 1.43122390610189 | 1.09377223666966 |
| Rural Residence | 1.21338691540353 | 1.10153843119681 |
| Smoking Status | 2.13821870292232 | 1.20924146566233 |
| Drinking status | 1.51970603029899 | 1.11029886713994 |
| Marital Status | 1.08715743658184 | 1.04266842120678 |
| Diabetes | 1.3104757968316 | 1.14476014816712 |
| Hypertension | 1.32223099897971 | 1.14988303708669 |
| Dyslipidemia | 1.13252513707227 | 1.06420164305092 |
| Kidney Disease | 1.01249014352132 | 1.00622569213935 |
| Liver Disease | 1.00991772936658 | 1.00494663010857 |
| Systolic Blood Pressure | 2.77873607124015 | 1.66695412991484 |
| Diastolic Blood Pressure | 2.51177369411647 | 1.58485762581895 |
| LDL Cholesterol | 2.97932461688976 | 1.72607201961267 |
| Total Cholesterol | 3.28318751115789 | 1.81195681823765 |
| HDL Cholesterol | 1.40861771302629 | 1.18685201816667 |
| HbA1c | 1.29876411696581 | 1.1396333256648 |
| TyG-WHtR | 1.7239503250301 | 1.09501682269232 |

Abbreviations: Adj_VIF: Adjusted Variance Inflation Factor. GVIF: Generalized Variance Inflation Factor. HbA1c: Glycated Hemoglobin A1c. TyG-WHtR: triglyceride glucose-waist height ratio.
