# Supplementary material for: Cumulative average triglyceride glucose-waist height index and incident cardiovascular disease in middle-aged and older adults: A nationwide cohort study from the china health and retirement longitudinal study
Source: PLoS One. 2026 Feb 26;21(2):e0333827. doi: 10.1371/journal.pone.0333827 (PMC12944753; doi:10.1371/journal.pone.0333827)
Supplement: S2 Table — (DOCX) [file pone.0333827.s003.docx]

 S2 Table. Association between the cumulative average TyG and CVD incidence

| Cumulative Average TyG | Quartiles | | | | | Continuous |
| --- | --- | --- | --- | --- | --- | --- |
|  | Quartile 1 | Quartile 2 | Quartile 3 | Quartile 4 | P for trend | Per 1 SD increase |
| Crude, OR (95% CI) | Reference | 1.124 (0.894–1.415) | 1.117 (0.888–1.406) | 1.169 (0.931–1.469) | 0.213 | 1.068 (0.987–1.155) |
| Model 1, OR (95% CI) | Reference | 1.086 (0.862–1.369) | 1.103 (0.874–1.391) | 1.159 (0.921–1.461) | 0.220 | 1.073 (0.989–1.162) |
| Model 2, OR (95% CI) | Reference | 1.051 (0.830–1.332) | 1.032 (0.808–1.320) | 1.064 (0.815–1.389) | 0.703 | 1.047 (0.949–1.154) |
| Model 3, OR (95% CI) | Reference | 1.038 (0.816–1.321) | 1.019 (0.791–1.312) | 1.011 (0.748–1.366) | 0.970 | 1.059 (0.931–1.204) |

Crude: No covariates were adjusted. Model 1, adjusted for age and gender; Model 2, adjusted for age, gender, smoking status, drinking status, SBP, DBP, HbA1c, HDL-c, LDL-c; Model 3, adjusted for all covariates. TyG-WHtR, triglyceride glucose-waist height ratio; CVD, Cardiovascular disease; OR, odds ratio; CI, confidence interval; SD, standard deviation.
