# Supplementary material for: Cumulative average triglyceride glucose-waist height index and incident cardiovascular disease in middle-aged and older adults: A nationwide cohort study from the china health and retirement longitudinal study
Source: PLoS One. 2026 Feb 26;21(2):e0333827. doi: 10.1371/journal.pone.0333827 (PMC12944753; doi:10.1371/journal.pone.0333827)
Supplement: S3 Table — (DOCX) [file pone.0333827.s004.docx]

 S3 Table. Association between the cumulative average WHtR and CVD incidence

| Cumulative Average WHtR | Quartiles | | | | | Continuous |
| --- | --- | --- | --- | --- | --- | --- |
|  | Quartile 1 | Quartile 2 | Quartile 3 | Quartile 4 | P for trend | Per 1 SD increase |
| Crude, OR (95% CI) | Reference | 1.223 (0.954–1.571) | 1.407 (1.105–1.797) | 1.961 (1.559–2.476) | <0.001 | 1.256 (1.162–1.362) |
| Model 1, OR (95% CI) | Reference | 1.240 (0.965–1.595) | 1.394 (1.089–1.788) | 1.815 (1.424–2.321) | <0.001 | 1.216 (1.125–1.319) |
| Model 2, OR (95% CI) | Reference | 1.210 (0.939–1.562) | 1.282 (0.992–1.660) | 1.608 (1.238–2.096) | <0.001 | 1.174 (1.087–1.276) |
| Model 3, OR (95% CI) | Reference | 1.211 (0.937–1.567) | 1.171 (0.902–1.522) | 1.429 (1.093–1.874) | 0.017 | 1.140 (1.057–1.237) |

Crude: No covariates were adjusted. Model 1, adjusted for age and gender; Model 2, adjusted for age, gender, smoking status, drinking status, SBP, DBP, HbA1c, HDL-c, LDL-c; Model 3, adjusted for all covariates. TyG-WHtR, triglyceride glucose-waist height ratio; CVD, Cardiovascular disease; OR, odds ratio; CI, confidence interval; SD, standard deviation.
