# Supplementary material for: Cumulative average triglyceride glucose-waist height index and incident cardiovascular disease in middle-aged and older adults: A nationwide cohort study from the china health and retirement longitudinal study
Source: PLoS One. 2026 Feb 26;21(2):e0333827. doi: 10.1371/journal.pone.0333827 (PMC12944753; doi:10.1371/journal.pone.0333827)
Supplement: S4 Table — (DOCX) [file pone.0333827.s005.docx]

 S4 Table. Association Between TyG-WHtR at Wave 1 and Incident CVD Incidence from Wave 2 to Wave 4: Cox Regression Results.

| Cumulative Average TyG-WHtR | Quartiles | | | | | Continuous |
| --- | --- | --- | --- | --- | --- | --- |
|  | Quartile 1 | Quartile 2 | Quartile 3 | Quartile 4 | P for trend | Per 1 SD increase |
| Crude, HR (95% CI) | Reference | 1.257 (1.074–1.472) | 1.362 (1.167–1.589) | 1.841 (1.590–2.131) | <0.001 | 1.253 (1.195–1.314) |
| Model 1, HR (95% CI) | Reference | 1.248 (1.065–1.462) | 1.308 (1.118–1.529) | 1.701 (1.461–1.982) | <0.001 | 1.214 (1.155–1.276) |
| Model 2, HR (95% CI) | Reference | 1.202 (1.026–1.409) | 1.177 (1.005–1.379) | 1.451 (1.240–1.698) | <0.001 | 1.146 (1.088–1.207) |
| Model 3, HR (95% CI) | Reference | 1.184 (1.008–1.391) | 1.132 (0.958–1.337) | 1.380 (1.147–1.661) | 0.0026 | 1.154 (1.076–1.237) |

Crude: No covariates were adjusted. Model 1, adjusted for age and gender; Model 2, adjusted for age, gender, smoking status, drinking status, SBP, DBP, HbA1c, HDL-c, LDL-c; Model 3, adjusted for all covariates. TyG-WHtR, triglyceride glucose-waist height ratio; CVD, Cardiovascular disease; HR, hazard ratio; CI, confidence interval; SD, standard deviation.
