# Supplementary material for: Cumulative average triglyceride glucose-waist height index and incident cardiovascular disease in middle-aged and older adults: A nationwide cohort study from the china health and retirement longitudinal study
Source: PLoS One. 2026 Feb 26;21(2):e0333827. doi: 10.1371/journal.pone.0333827 (PMC12944753; doi:10.1371/journal.pone.0333827)
Supplement: S5 Table — (DOCX) [file pone.0333827.s006.docx]

S5 Table. Association between the cumulative average TyG-WHtR and CVD incidence after excluding individuals with any missing value

| Cumulative Average TyG-WHtR | Quartiles | | | | | Continuous |
| --- | --- | --- | --- | --- | --- | --- |
|  | Quartile 1 | Quartile 2 | Quartile 3 | Quartile 4 | P for trend | Per 1 SD increase |
| Crude, OR (95% CI) | Reference | 1.482 (1.117–1.974) | 1.784 (1.358–2.358) | 2.071 (1.584–2.725) | < 0.001 | 1.318 (1.206–1.441) |
| Model 1, OR (95% CI) | Reference | 1.505 (1.131–2.010) | 1.753 (1.326–2.330) | 1.965 (1.485–2.617) | < 0.001 | 1.289 (1.174–1.415) |
| Model 2, OR (95% CI) | Reference | 1.487 (1.110–1.999) | 1.657 (1.232–2.238) | 1.803 (1.307–2.500) | < 0.001 | 1.257 (1.125–1.405) |
| Model 3, OR (95% CI) | Reference | 1.475 (1.099–1.987) | 1.541 (1.139–2.094) | 1.579 (1.122–2.231) | 0.014 | 1.228 (1.087–1.388) |

Crude: No covariates were adjusted. Model 1, adjusted for age and gender; Model 2, adjusted for age, gender, smoking status, drinking status, SBP, DBP, HbA1c, HDL-c, LDL-c; Model 3, adjusted for all covariates. TyG-WHtR, triglyceride glucose-waist height ratio; CVD, Cardiovascular disease; OR, odds ratio; CI, confidence interval; SD, standard deviation.
